# Generation of a new Paneth cell–specific *Cre*-recombinase transgenic mouse line

**DOI:** 10.3389/fimmu.2025.1576995

**Published:** 2025-06-25

**Authors:** Natalia Garcia-Gonzalez, Charlotte Wallaeys, Wendy Toussaint, Tino Hochepied, Sylviane Dewaele, Somara De Beul, Robin Van Loke, Jolien Vandewalle, Steven Timmermans, Richard S. Blumberg, Claude Libert

**Affiliations:** 1 VIB Center for Inflammation Research, Ghent, Belgium; 2 Department of Biomedical Molecular Biology, Ghent University, Ghent, Belgium; 3 Division of Gastroenterology, Department of Medicine, Brigham and Women’s Hospital, Harvard Medical School, Boston, MA, United States

**Keywords:** Paneth cells, *Defa24iCre*, defensins, *Cre*-recombinase transgenic mouse model, *Cre/loxP*

## Abstract

Paneth cells are located in the crypts of Lieberkühn in mammalian small intestines and are producing antimicrobial peptides to keep the microbiome under control. The genetic manipulation of Paneth cells and their tracking and depletion depend on a solid Paneth cell–specific *Cre*-transgenic line. Here, we describe bulk RNA sequencing (RNA-seq)–based expression data from pure, sorted Paneth cells of C57BL/6J mice and identify several strongly expressed Paneth cell–specific genes, the expression of which is stable under pathophysiological conditions, as well as in the duodenum, jejunum, and ileum. We selected the *Defa24* gene regulatory sequences and generated a new *Defa24iCre* transgenic line using BAC technology, Tg(Defa24-icre)Cli. The resulting transgenic line provides robust expression and allows for the complete depletion of Paneth cells by cell ablation, yielding mice without any detectable lysozyme biological activity in the small intestines.

## 1 Introduction

Paneth cells are highly differentiated, long-lived intestinal epithelial cells. They are found in the crypts of Lieberkühn, in the small intestine (duodenum, jejunum, and ileum) of mammals (1, 2). Paneth cells express markers that allow them to be sorted by fluorescence-activated cell sorting (FACS). Although single-cell and bulk RNA sequencing (RNA-seq) studies have found differences between PCs across these three regions of the murine small intestine (3, 4), their gene expression profile clearly reveals their major overall functions: (i) they produce and secrete antimicrobial peptides (AMPs), (ii) they produce proteins and metabolites to keep the stemness of neighboring stem cells, and (iii) they regulate the uptake of heavy metals such as Zn^2+^ (1). Despite isolation of Paneth cells leading to 99.99% pure Paneth cell populations, which can be analyzed by RNA-seq and mass spectrometry (3, 5), their culture and genetic manipulation remain points of improvement. The quantitative RNA-seq data recently obtained from sorted Paneth cells (1, 6–8), combined with mouse cell atlas data (9–11), shed light on the Paneth cell specificity and quantity of expression of their genes. These may provide inspiration to generate new, improved transgenic tools.

As generally appreciated, cell type–specific *Cre* transgenic mice can be applied not only to generate cell-specific knockout alleles but also transgenic overexpressions (12). The latter is achieved by crossing cell-specific *Cre* transgenic mice with mice carrying transgenes that become active by Cre activity, that is, by removing “floxed” STOP cassettes (13). Examples include the cell type–specific expression of diphteria toxin A (DTA), leading to targeted cell ablation, or the expression of reporter genes, such as TdTomato, labeling the *Cre*-expressing cells in red (14). Promoters driving *Cre* expression should be cell-specific, robustly active in all target cells, and stable over time. Ideally, their activation should not be influenced by external factors such as inflammation or hormonal changes (5, 15).

Paneth cell–specific transgenic mice have been described in the past. The first Paneth cell–specific transgene, created in 1997 by Garabedian and colleagues, used the mouse homologue of the human *cryptdin-2* gene promoter, named by the authors the *cryptdin-2* promoter. This promoter, described as the – 6500 bp to +34 bp fragment of the *cryptdin 2* gene, was used to drive the expression of the DTA gene, leading to an 82% reduction of Paneth cells in transgenic mice (16). This approach proved to be more specific and durable compared to earlier methods to deplete Paneth cells, such as dithizone injection (17–19). Eleven years later, Vaishnava et al. (2008) used the transgene plasmid created by Garabedian et al. to create a *Cryptdin-2* promoter-driven *Myd88* gene, specifically in Paneth cells (20). These tools did not involve Cre recombinase.

The first Paneth cell–specific *Cre*-expressing mice were created by Adolph et al. in 2013, building upon the transgene plasmid created in 2007 (21). Here, the authors defined the *cryptdin 2* sequence as *Defa6* in mice and placed the improved *Cre* gene, *iCre*, under its control. The construct was injected into zygotes and integrated randomly in the genome. Functional work with this iCre line yielded groundbreaking data and supported its Paneth cell–specific expression of *iCre* (5, 21). However, it was found that the iCre activity was low; for example, Paneth cell–specific removal of a simple floxed allele of *Tnfrsf1a* was only partial. Even when the Defa6 promoter-driven *iCre* was bred to homozygosity, reduction of Tnfrf1a expression and function was only around 50%–60% (5).

Regarding Paneth cell–specific *Cre*-expressing mice, in 2018 Burger et al. developed a *Defa4-*promoter-driven *Cre* mouse by knocking in the *Cre* gene in the 3’UTR of the *Defa4* gene. The construction yields bi-cistronic mRNAs, from which DEFA4 protein is translated, as well as CRE protein, thanks to an internal ribosome entry site (IRES) (22). The *Cre* expression is Paneth cell–specific, but the genome annotations of *Defa* genes have been revised, and *Defa4* is not present in the current annotation, but the official synonym, *Defa28*, according to RefSeq records, is.

In 2019, Van Es et al. created a *Cre-ERT2* knock-in at the ***Lyz1*** locus (23), thereby inactivating the *Lyz1* allele. Yu et al. (2018) also generated a similar mouse, again with inactivation of the *Lyz1* allele (24). The *Lyz1* gene is strongly expressed in Paneth cells, and inactivation of one allele may influence Paneth cell physiology, thereby requiring the use of such a *Cre* mouse model exclusively in a heterozygous condition. Also, the mouse cell atlas and a recent paper reveal that *Lyz1* might not be a Paneth cell–specific expressed gene but also shows expression in alveolar macrophages (9, 10, 25). In 2023, Balasubramanian et al. reported the construction of a *Lyz1* promoter-*Cre* BAC construct, which was used to generate transgenic mice (26), in which the BAC clone had integrated randomly in the mouse genome. This will not inactivate one of the *Lyz1* alleles.

In this study, we considered the existing Paneth cell–specific *Cre* transgenic mouse lines and studied specificity, strength of expression, and stability, and we decided to generate an improved Paneth cell–specific *Cre* transgenic mouse using a *Defa24* gene-containing BAC strategy. We give an overview of the expression strength and specificity of this new tool, the Tg(Defa24-icre)Cli line, and use it to create a full PC ablation line based on cell type-specific DTA expression.

## 2 Materials and methods

### 2.1 Mice

All mice used in this study were housed in individually ventilated cages within a specific pathogen-free (SPF) animal facility, maintained under a 14/10h light/dark cycles, and received food and water *ad libitum.* All mice were used at the age of 8–12 weeks, and all experiments were approved by the institutional ethics committee for animal welfare of the Faculty of Sciences, Ghent University, Belgium.

### 2.2 *Defa6iCre* transgenic mice

*Defa6iCre* transgenic mice were generated and described by Adolph et al. (21) and generously provided by Dr. Blumberg (5). They were maintained on a C57BL/6J background. The official name of the line is Tg(Defa6-icre)Rsb.

### 2.3 Generation of *Defa24iCre* transgenic mice

The *iCre* gene, followed by an Frt-kan/neo-Frt selection cassette, was recombined in the RP24-251F2 BAC clone, containing the mouse *Defa24* locus, and replaced the *Defa24* coding sequence via Red/ET recombination in *E. coli*. The selection cassette was removed by expression of *Flpe* in *E. coli* and the modified BAC were digested with the ClaI restriction enzyme. The 42 kB fragment containing the modified *Defa24* locus was isolated from gel using PFGE followed by electro-elution and was purified via Amicon ultra 100 kD centrifugation (Millipore Corporation, Billerica, MA). The fragment was injected at 1 ng/µl into the pronucleus of 147 C57BL/6J zygotes, and the zygotes were transferred into the oviduct of pseudopregnant recipient females, resulting in 14 live offspring, in 4 of which (founders) the modified BAC construct was randomly integrated in the genome. Based on this procedure, the official nomenclature of the mouse line is called Tg(Defa24-icre) Cli. Genotyping of the *Defa24iCre* transgenic construct in mouse gDNA was done using primers listed in the **Supplementary Table S2**, including *A20* as an internal control.

### 2.4 TdT and DTA transgenic mice

TdTomato reporter mice have been described elsewhere (27). Briefly, they harbor a TdT cDNA expression cassette under control of the *Rosa26* promoter but preceded by a floxed STOP sequence. Similarly, DTA mice have been described elsewhere (28). Briefly, they harbor a DTA cDNA expression cassette under control of the *Rosa26* promoter, but intermitted by a floxed STOP sequence. The mutation is kept in C57BL/6J mice.

To generate Paneth cell–specific TdT-expressing mice, *TdT ^fl/w^*^t^ reporter mice (which have the *TdT* gene under control of the ROSA26 promotor but interrupted by a floxed STOP cassette) were crossed with *Defa6iCre*^Tg/+^ and with *Defa24iCre*^Tg/+^ mice, yielding *TdT^Defa6iCre^*^Tg/+^ and *TdT^Defa24iCrTg/+^* mice (which have one TdT^fl^ allele and one iCre allele) as well as TdT^Tg/+^ mice (which have one TdT^fl^ allele but no iCre allele). To generate Paneth cell–specific DTA-expressing mice, essentially, a similar breeding scheme was developed.

### 2.5 Paneth cell sorting and RNA-seq

We have recently described the protocol to sort Paneth cells from mice in detail (5, 6, 8). The sorted Paneth cells are 99.9% pure, and a single C57BL/6J mouse yields, on average, some 25,000 Paneth cells after sorting. We thus obtained RNA-seq data comprising over 30,000 different transcripts per sample and millions of reads (3). Paneth cells were FACS-enriched by sorting CD24^+^-cKIT^+^- SSC^Medium – High^ - CD31^−^ CD45^−^ TER119^-^ cells and collected in 1 ml sorting buffer (50% advanced DMEM/F-12 and 50% FCS). Paneth cell RNA isolations were performed using the RNeasy Plus Micro Kit Qiagen (Qiagen Belgium, Antwerp, Belgium) according to manufacturing conditions.

The RNA was used for creating an Illumina sequencing library using the Illumina TruSeqLT stranded RNA-seq library protocol (VIB Nucleomics Core), and single-end sequencing was done on the NovaSeq 6000. The obtained reads were mapped to the mouse reference transcriptome/genome (mm39/gencode v28) with STAR (2.7.10a), and read counts were obtained during alignment using the STAR”–quantMode GeneCounts” option. Differential gene expressions were assessed with the DESeq2 package, with the False Discovery Rate (FDR) set at 5%. RNA-seq data deposited at the National Center for Biotechnology Information Gene Expression Omnibus public database (http://www.ncbi.nlm.nih.gov/geo/) under the following accession numbers:

○ Paneth cells sorted from wild-type C57BL/6J mice, (*n* = 3): GSE269510 (**Supplementary Table S1**).
○ Paneth cells from C57BL/6J mice, either injected with 250 μl PBS or injected with 25 μg recombinant TNF (*n* = 4): GSE267790 (**Table 1**).
○ Paneth cells sorted from wild-type C57BL/6J mice, (*n* = 4), namely, from the duodenum, the jejunum, and the ileum: GSE255507 (**Table 2**).

**Table 1:** Gene expression values of Paneth cell–specific transcripts.

Gene	PBS_AVG	PBS_SD	TNF_AVG	TNF_SD	LFC	Padj
*Lyz1*	1681604	322126	2062555	514008	0.294594	0.163262
*Defa24 (=Defa6)*	1245968	371616	1078165	291453	−0.208689	0.610567
*Defa30*	537285	182944	415349	132368	−0.371363	0.428168
*Defa38*	406747	128023	186154	50520	−1.127639	0.001286
*Defa39*	294969	122104	66675	24263	−2.145352	0.000002
*Defa22*	100967	43074	340845	106962	1.755229	0.000046
*Defa21*	72849	41827	259165	87673	1,830906	0.000685
*Defa29*	53940	21008	37698	11901	−0.516893	0.279306
*Defa17*	12114	7991	4237	2089	−1.515624	0.087261
*Defa26*	10788	4195	5560	1813	−0.956297	0.032021
*Defa40*	10309	3898	5309	1697	−0.957254	0.027381
*Defa3*	3076	1673	1362	464	−1.175205	0.067989
*Defa35*	1875	363	8395	1603	2.162759	0.000000
*Defa5*	949	392	1770	608	0.899837	0.060816
*Defa28 (=Defa4)*	836	310	114	43	−2.869948	0.000000
*Defa34*	507	187	1172	500	1.209504	0.010515
*Defa41*	348	151	3921	811	3.495428	0.000000
*Defa43*	71	22	52	14	−0.425497	0.348928
*Defa42*	67	16	91	25	0.428048	0.283093
*Defa27*	33	5	8	4	−1.966498	0.002878
*Defa25*	17	5	7	6	−1.183827	0.228820
*Defa37*	2	2	2	1	0.071206	0.981592
*Defa23*	1	1	0	1	−0.892060	1.000000
*Defa32*	0	1	0	0	−0.885442	1.000000
*Defa31*	0	1	1	2	1.450229	1.000000
*Defa2*	0	0	0	0	0.000000	1.000000
*Defa20*	0	0	0	0	0.000000	1.000000
*Defa33*	0	0	0	0	0.000000	1.000000
*Defa36*	0	0	0	1	1.479204	1.000000

Workflow: C57BL/6J mice received a challenge with 25 μg TNF or PBS. 15h later Paneth cells were sorted and subjected to RNA-seq (*n* = 4). Column A is the genes of the transcripts. Columns B and D are mean values of the RNA-seq expression, and C and E represent standard deviations (SDs). Columns F and G represent the log(2) fold change of the values of D *versus* B, and Column G the associated adjusted *P*-value. Shadowed cells indicate significant variations in gene expression based on the *P*-value.

**Table 2:** Gene expression values of Paneth cell–specific transcripts in duodenum, jejunum, and ileum.

Gene	AVG_D	SD_D	AVG_JE	SD_JE	AVG_IL	SD_IL	LFC D-JE	*P*adj D-JE	LFC D-IL	*P*adj D-IL	LFC JE-IL	*P*adj JE-IL
*Defa24* (=*Defa*6)	2109742	691159	2886508	543841	1727992	609222	−0.45226	0.59928	0.28797	0.64915	0.74023	0.17908
*Lyz1*	1817088	584303	2119920	318031	1668282	488337	−0.22238	0.80885	0.12326	0.85652	0.34565	0.68717
*Defa30*	1122852	399953	1240373	250052	778620	291728	−0.14361	0.90816	0.52818	0.33793	0.67178	0.31276
*Defa21*	14770	4780	169909	38861	495802	173828	−3.52414	0.00000	−5.06914	0.00000	−1.54500	0.00004
*Defa22*	12553	3751	151392	32274	484897	158012	−3,59234	0.00000	−5.27174	0.00000	−1.67940	0.00000
*Defa38*	539583	147438	654951	113222	283349	86365	−0.27955	0.74163	0.92926	0.00703	1.20881	0.00039
*Defa39*	509402	143455	670117	113990	158742	51745	−0.39561	0.61670	1.68213	0.00000	2.07774	0.00000
*Defa29*	331335	7825	91640	16305	89266	23419	−1.46768	0.00000	−1.42981	0.00000	0.03787	0.98194
*Defa17*	76941	25353	90950	14937	53343	17713	−0.24133	0.80251	0.52846	0.27512	0.76979	0.12366
*Defa34*	4757	1398	13663	3023	31504	12676	−1.52228	0.00016	−2.72758	0.00000	−1.20530	0.00392
*Defa5*	5101	1823	19477	4322	25451	9714	−1.93328	0,00000	−2.31918	0.00000	−0.38591	0.72079
*Defa26*	29696	10980	43593	8612	21651	7927	−0.55389	0.51253	0.45583	0.43072	1.00971	0.03619
*Defa40*	10388	4308	26421	6264	12829	5144	−1.34691	0.06496	−0.30459	0.75093	1.04232	0.18255
*Defa3*	6895	2597	11183	2409	7163	2763	−0.69784	0.37914	−0.05508	0.95139	0.64276	0.36878
*Defa35*	274	91	1603	323	5006	1857	−2.55555	0.00000	−4.19809	0.00000	−1.64254	0.00001
*Defa41*	79	32	679	256	1824	882	−3.11916	0.00000	−4.54451	0.00000	−1.42535	0.07671
*Defa28 (=Defa4)*	671	227	1039	171	904	358	−0.63298	0.42316	−0.43148	0.46058	0.20150	0.90103
*Defa42*	9	3	35	9	86	35	−1.98422	0,00096	−3.29176	0.00000	−1,30754	0.01918
*Defa27*	30	16	47	8	56	18	−0.68168	0,57868	−0.93938	0.16019	−0.25770	0.90848
*Defa43*	41	8	59	20	43	16	−0.54497	0,58987	−0.09809	0.91850	0.44688	0.68936
*Defa25*	11	4	21	7	41	10	−0.95663	0,38671	−1.97223	0.00015	−1.01560	0.18255
*Defa32*	1	2	1	1	22	10	0.20881	0,97086	−4.49822	0.00032	−4.70703	0.00182
*Defa33*	0	0	1	1	16	6	−1.78315	0,71749	−6.58763	0.00000	−4.80448	0.00234
*Defa31*	4	2	10	2	3	2	−1.22498	0,44736	−0.49359	0.76223	1.71857	0.15930

Paneth cells were sorted from duodenum (D), jejunum (JE), and Ileum (IL) and subjected to RNA-seq (*n* = 4). Column A is the genes of the transcripts. Columns B, D, and F are mean values of the RNA-seq expression, and C, E, and G represent standard deviations (SDs). Columns H, J, and L represent the log(2) fold change of the values of the means and SDs, and Column I, K, and M the associated adjusted *P*-values. Shadowed cells indicate significant variations in gene expression based on the *P*-value.

### 2.6 Tissue processing and immunohistochemistry

After sampling, tissue was fixed overnight at 4°C in 4% PFA. After transferring to 70% ethanol, ileums were dehydrated within the Shandon Citadel 2000 (Thermo Fisher Scientific) and bedded in paraffin. For hematoxylin and eosin staining (H&E) and Alcian Blue/PAS staining, paraffin blocks were sectioned at 5 μm using the HM340E Semi-automated microtome (Thermo Fisher Scientific) and further dewaxed and stained using the Leica ST5010 Autostainer XL. For immunohistochemistry, paraffin blocks were sectioned at 5 μm using the HM340E Semi-automated microtome (Thermo Fisher Scientific). Deparaffinization and rehydration were performed in the Leica ST5010 Autostainer XL. The sections were then washed with PBS, and antigen retrieval was performed with proteinase K in Ca-TE buffer (1/20) (15 min at 37°C). Five percent of donkey serum was diluted (1/100) in PBST (PBS + 0.5% BSA + 0.1% Tween20) and used as a blocking solution [30 min at room temperature (RT)]. Polyclonal rabbit anti-human lysozyme (LYZ1, A0099, Agilent) in PBST (1/1000) was used as the primary antibody (incubated overnight at 4°C), and after washing (PBS), donkey anti-rabbit Alexa Fluor^®^ 568 (A10042, Invitrogen) in PBST (1/500) was used as the secondary antibody (1h at RT). Tissue was counterstained with DAPI (D1306, Thermo Fisher Scientific, 1/1000 in PBS) for 15 min at RT. Slides were then mounted with polyvinyl alcohol + DABCO (Sigma). To test for nonspecific binding of the secondary antibody, a negative control was made per sample by leaving the blocking solution on the sample instead of adding the primary antibody. TdT staining was performed on cryosections. Cryoblocks were sectioned at 10 μm using the CryoStar NX70. Slides were air-dried for 15 min at RT and washed with PBS. The same protocol was used as previously described for LYZ1, starting from the serum step, but here 5% goat serum was used. Polyclonal rabbit anti-RFP (600-401-379, Rockland) in PBST (1/1000) was used as the primary antibody, and goat anti-rabbit Alexa Fluor^®^ 568 (A-11011, Invitrogen) in PBST (1/500) was used as the secondary antibody. Microscopic images were taken with the Zeiss Axioscan 7 microscope.

### 2.7 Real-time quantitative PCR

Ileum was isolated, put in RNA later (Life Technologies Europe), and stored at −20°C before RNA was isolated. Total RNA was isolated with the Aurum™ total RNA mini kit (7326820, Bio-Rad) according to the manufacturer’s instructions. RNA concentration was measured with the Nanodrop 8000 (Thermo Fisher Scientific), and 1,000 ng RNA was used to prepare cDNA with the Sensifast cDNA Synthesis Kit (Bioline). cDNA was diluted 10 times in ultrapure water for use in RT-qPCR reactions. RT-qPCR primers for used targets are listed in **Supplementary Table S3**. The RT-qPCR reaction was performed with SensiFast Sybr no-ROX mix (Bioline) and was performed in duplicate in a Roche LightCycler 480 system (Roche). The stability of the housekeeping genes (HKGs) was determined using the geNorm House Keeping Gene Selection Software from QBase (Biogazelle). Results are given as relative expression values normalized to the geometric mean of the HKGs, calculated in the qBase+ software (version 3.4, Biogazelle, Ghent).

## 3 Results

### 3.1 Tracing back the origins of the existing Paneth cell transgenic mice constructs and promoters

As mentioned above, the lysozyme 1 (*Lyz1*) and the alpha defensin coding genes, the *Defa* genes, are considered Paneth cell–specific and hence candidates to consider for transgenic strategies. In mice, all *Defa* genes are found in one single locus on chromosome 8. The *Defa* locus in laboratory mouse strains, such as the reference strain C57BL/6J, is complex and considerably different when consulting the three major mouse genome browsers, namely those from Mouse Genome Informatics, Refseq, and Gencode (3). Based on a detailed comparison of these three databases and incorporation of our Paneth cell bulk RNA-seq and single-cell data, we have recently investigated and updated the current status of the *Defa* locus (3).

**Figure 1:**
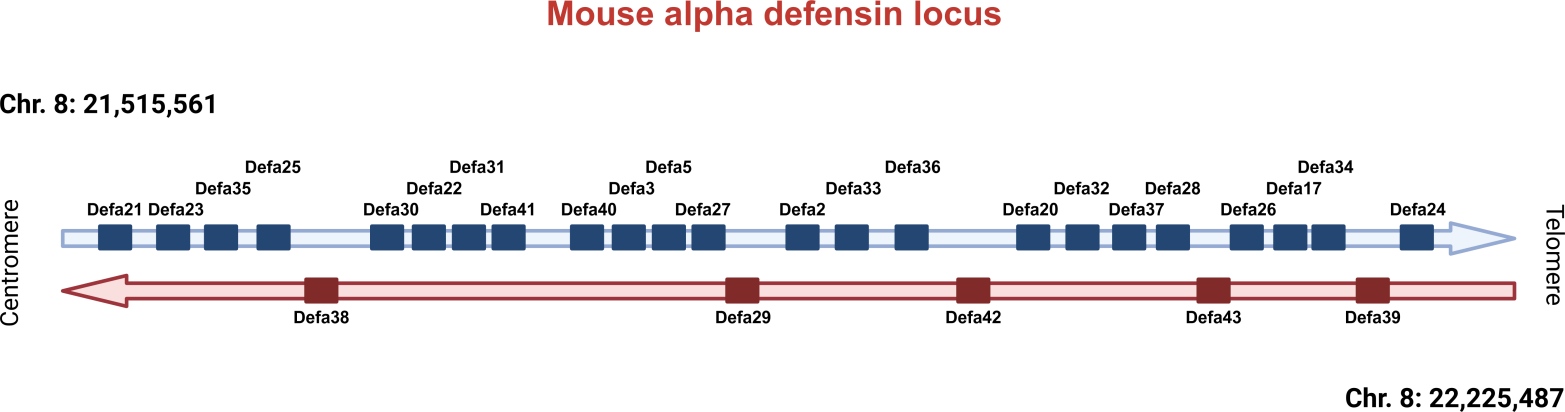
Overview of the *Defa* locus in mice. Current status of the *Defa* locus on mouse chromosome 8, based on recent research. Centromeric direction on the left, telomeric on the right. *Defa* genes are transcribed from the horizontal strand (in blue) or from the reverse horizontal strand (in red). The distances between genes are not drawn in scale, but can be found in Timmermans et al., 2025 (3).

Mice have 28 protein-coding *Defa* genes in their genome (**Figure 1**). During recent updates, genes have been renamed or deleted because they proved to be redundant or nonexistent. In fact, *Defa4* no longer exists. Upon investigation of the *Defa4* promoter sequences used to generate the *Defa4-Cre* transgenic animals, as well as the historical and current RefSeq records, it is highly likely that the authors applied *Defa28* sequences to generate the knock-in of *Cre* recombinase and that the *Defa4*-*Cre* transgenic mice are in fact *Defa28*-*Cre* transgenic mice.

Similarly, the *Defa6* gene is no longer considered to exist because it appeared to refer to a gene sequence highly overlapping, in fact, identical to the *Defa24* gene. Hence, the *Defa6iCre* transgenic mice, generated by Adolph et al. (21) and also used by us in the past and in the study here described, were generated by cloning a DNA fragment of −6500 to +34 of the presumed *Defa6* gene and were in fact made by *Defa24* sequences. Here, we will use the old name (*Defa6iCre*) to avoid confusion. When tracing back the cloning strategy of the so-called *Cryptdin-2-*DTA and *Cryptdin-2-*Myd88 transgenic mice described in the introduction, both papers applied the same −6500 to +34 bp promoter fragment, identified by them as the mouse orthologue of *Cryptdin-2*, but it is in fact identical to the *Defa6* fragment applied by Adolph et al. and thus *Defa24* as well.

The mouse lines based on Lyz1 *Cre* transgenic lines use a different approach and have, as a primary disadvantage, that the endogenous *Lyz1* gene is disrupted by the *iCre* insert. Considering the role and abundance of *Lyz1* expression in PCs, this is likely to have a direct impact on Paneth cell function, microbial composition, and intestinal homeostasis.

### 3.2 Expression study of *Defa* genes and their stability in mice

Over the previous years, we have sorted 150 samples from C57BL/6J mice, each containing 10,000 or more Paneth cells, and performed bulk RNA-seq on them. We are displaying a typical example of such a bulk RNA-seq experiment in **Supplementary Table S1**. It concerns the expression values of transcripts in pure Paneth cell populations of wild-type C57BL/6J mice, ranked from the highest to the lowest detectable transcripts, that is, 13,130 different transcripts. *Lyz1* and some of the (correctly annotated) *Defa* genes are found on top of the list of most abundant mRNAs (*Defa24, Defa30, Defa38, Defa39, etc.*), and other *Defa* transcripts are only mildly expressed (*e.g., Defa28*) or low to very low (*Defa43, Defa27, Defa25, Defa31*).

We have studied the impact of antibiotics, hormones, drugs (such as injection of dexamethasone), and inflammation to study the stability of *Lyz1* and all *Defa* gene expressions in Paneth cells. We found that mosttreatments of mice had little impact, except acute intestinal inflammation, induced 15h after injection of the cytokine TNF (5). In **Table 1**, the expression values of *Lyz1* and 11 *Defa* (expressed) genes are not affected by TNF (including *Defa24*). However, for another 11 expressed *Defa* genes, their expression is significantly affected by acute inflammation, with five genes increasing in expression and six genes showing decreased expression, including *Defa28* (=*Defa4*), which decreases by over sevenfold. It may be expected that a *Defa4*-*Cre* (=*Defa28*-*Cre*) transgenic construct will suffer from such inflammation too.

### 3.3 Differences in three regions of the small intestine

Recently, we have studied regional differences of Paneth cell transcriptomes, using single-cell RNA-seq on sorted Paneth cells of the full small intestine and bulk RNA-seq of Paneth cells sorted from the duodenum, jejunum, and ileum (4). When focusing on *Lyz1* and the *Defa* genes that showed detectable expression (see **Table 2**), we found that ten genes had no significant differences in expression, per cell, in the three regions of interest: *Lyz1, Defa24, Defa30, Defa17, Defa40, Defa3, Defa28, Defa27, Defa43*, and *Defa31*. All other 14 genes displayed significant differences between Paneth cells of different regions, making these genes less suited to act as general Paneth cell *Cre* transgenic promoters. *Defa21* and *Defa22*, for example, are strongly expressed in ileum Paneth cells but very significantly less in duodenal and jejunal Paneth cells.

Based on the strength of expression (**Table 1**; **Supplementary Table S1**), the response to inflammation (**Table 1**), and regional differences (**Table 2**), *Lyz1, Defa24, Defa30*, and perhaps *Defa17* appear as the only conceivable options to drive *Cre* in a sufficient, stable, and spatially solid way.

### 3.4 Paneth cell specificity of expression

In the context of the intestine, all *Cre* transgenic lines used are highly Paneth cell–specific. In the small intestine of mice, *Lyz1* is a Paneth cell marker. However, single-cell experiments using other mouse tissues have shown that other cell types, outside of the intestine, also express *Lyz1*, even to the point where it can be used to assign cell identity. The mouse cell atlas shows that 2 cell types express *Lyz1* as a differentiating marker gene, namely a cluster of monocytes and of alveolar type II cells (https://bis.zju.edu.cn/MCA/atlas2.html) (9, 11, 29). *Defa24* and *Defa28* appeared to have no expression beyond Paneth cells. The ectopic expression of *Lyz1* (and therefore, highly likely *Lyz1-Cre*) in these cells may be problematic in certain experimental settings, leading to unpredictable off-target effects.

### 3.5 Generation of a new *Defa24-Cre* transgenic mouse line

We used the *Defa6iCre* line to generate tissue-specific KO lines and try to ablate Paneth cells using cell-specific DTA expression. However, the *Defa6iCre* was unable to mediate full ablation of Paneth cells, even with two copies of *Defa6-iCre*, thus in homozygous condition. This formed the main impetus to create a new iCre line with improved expression and penetrance of the *iCre* transgene.

**Figure 2:**
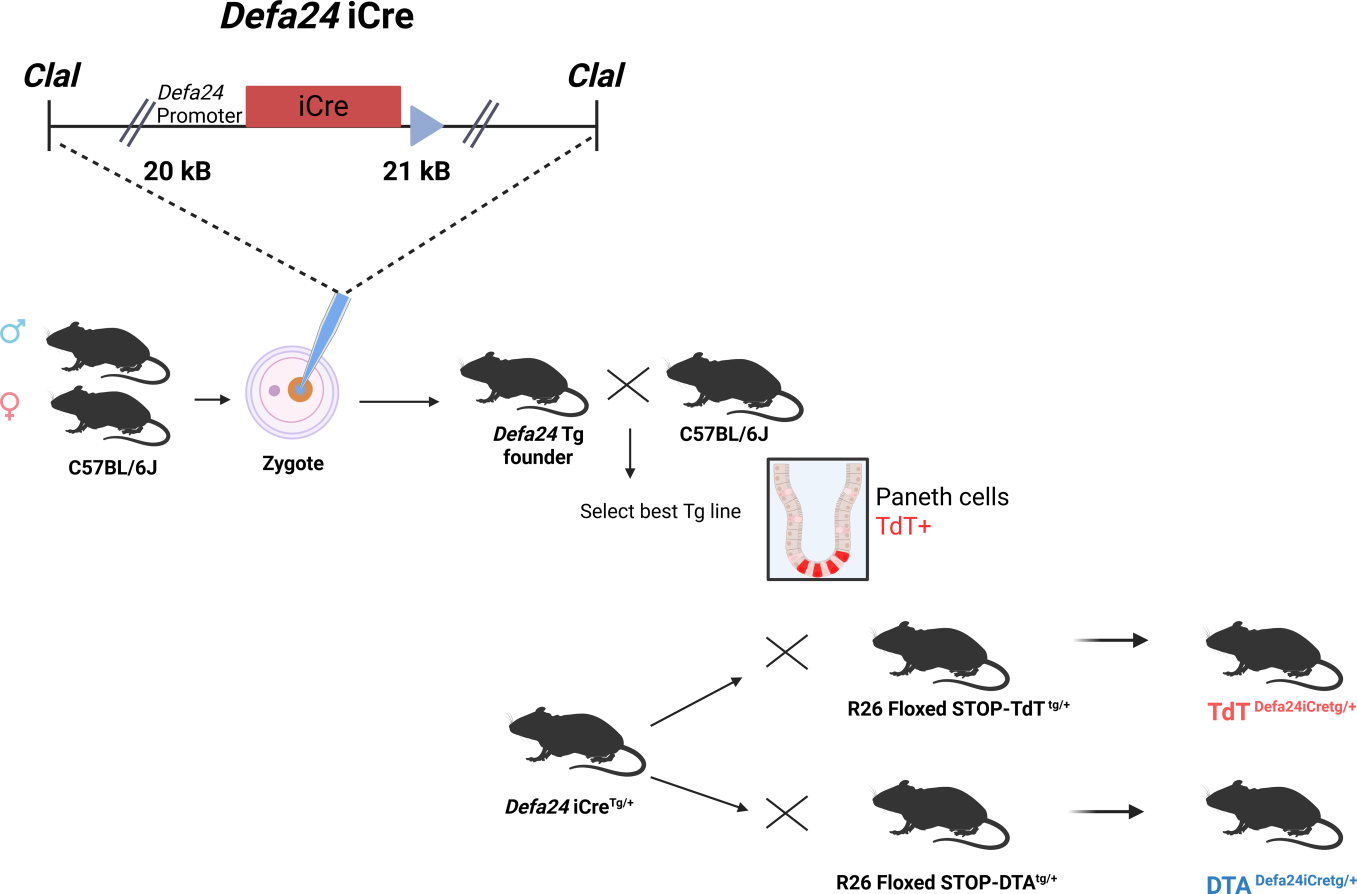
Overview of generation of *Defa24iCre* transgenic mice. The BAC insert, containing 20 kB upstream and 21 kB downstream of the coding unit of *Defa24*, was modified, since *Defa24* coding sequence was replaced by the *iCre* gene. The resulting 42 kB fragment was purified and injected in C57BL/6J zygotes, and four transgenic founders identified and crossed to select the best, based on TdT expression after crossing to TdT*^Defa24iCre^*^Tg/+^. Then, this line was compared with the TdT*^Defa6iCre^*^Tg/+^ mice, described before, at level of intensity of TdT signals. Also, with both Defa transgenic lines, mice were generated which were DTA*^Defa24iCre^*^Tg/+^ and compared to DTA*^Defa6iCre^*^Tg/+^ and DTA*^Defa6iCre^*^Tg/Tg^ mice, in terms of Paneth cell ablation.

As described in the Materials and **Supplementary Figure S1**, we inserted the *iCre* gene into the coding region of the C57BL/6J *Defa24* gene in a BAC clone, thereby replacing the *Defa24* coding region. The BAC was cut using *ClaI*, yielding a 42 kB construct containing 20 kB upstream and 21 kB downstream of the *Defa24* gene, regions that contained no other genes. This BAC clone was injected into zygote pro-nuclei, and four transgenic founder lines were identified. All four lines were crossed with C57BL/6J and yielded four heterozygous families, all 4 of which were crossed with R26 floxed STOP TdT^Tg/+^ mice, and the expression pattern of TdT was evaluated on tissue sections using anti-TdT antibody (**Figure 2**). One transgenic line had no TdT expression, and two lines had strong Paneth cell expression of TdT but displayed expression outside of the crypts, in the intestinal epithelium and in the submucosa (data not shown). One transgenic line, however, yielded a solid TdT expression selectively in Paneth cells. This transgenic line was chosen for further investigation. Differences found in *Cre* expression among the different founders can be related to the chromosomal context of the integration (30).

### 3.6 Comparison of *Defa24iCre* and *Defa6iCre* transgenic lines

**Figure 3:**
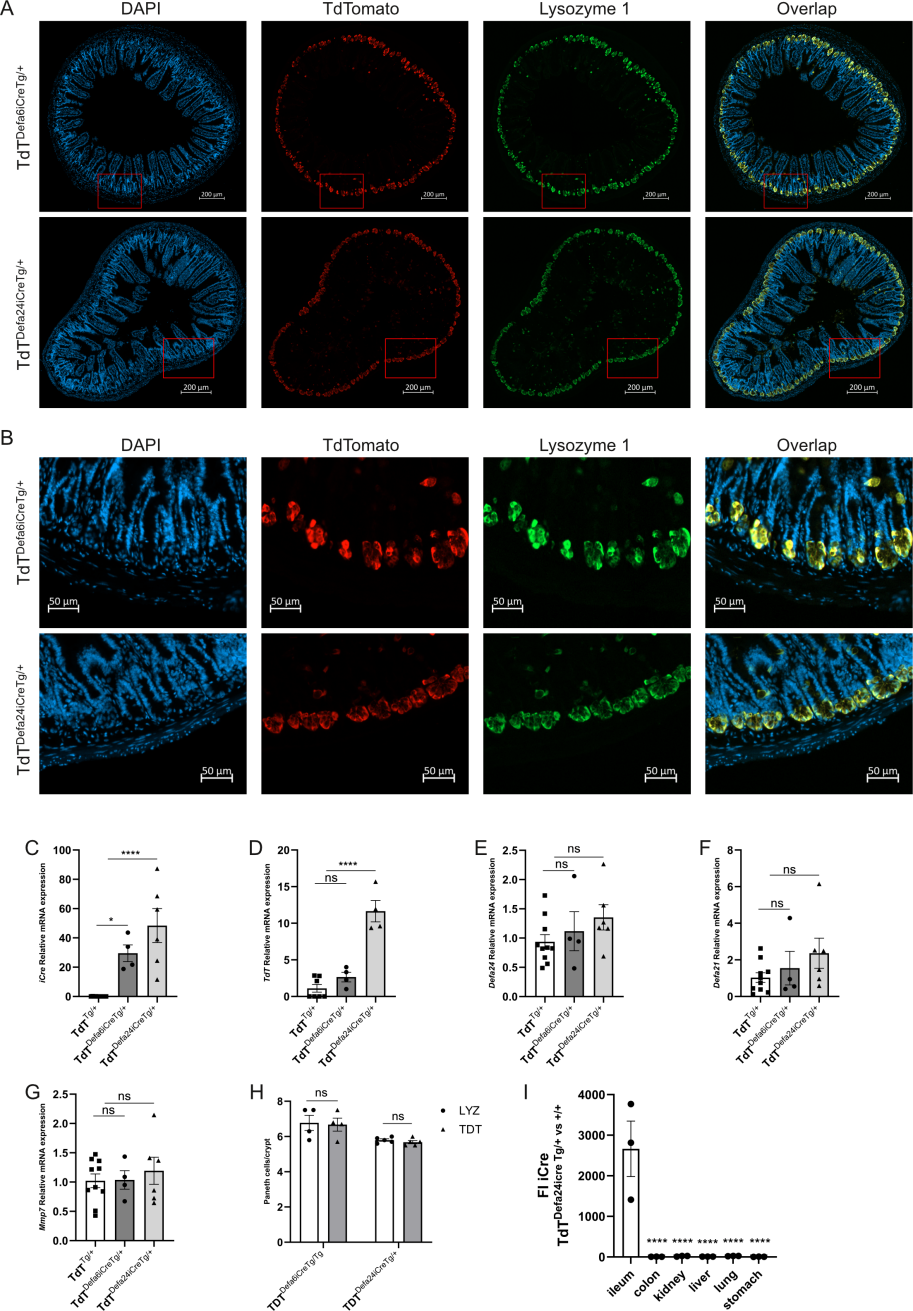
Comparison of cell type specificity and intensity of *iCre* expression in two transgenic lines. TdT*^Defa24iCre^*^Tg/+^ mice and TdT*^Defa6iCre^*^Tg/+^ mice were investigated at the age of 8 weeks. **(A)** Staining of ileum sections with DAPI, anti-TdT antibody, and anti-LYZ1 antibody and overlay of both pictures. Pictures taken with the Zeiss Axioscan 7 microscope (*n* = 3 and 4). **(B)** Zoomed in section cut-out of the images seen in **(A)**, providing a detailed view of the location of the TdT and LYZ1 signals. **(C–G)** From TdT*^Defa24iCre^*^Tg/+^ and TdT*^Defa6iCre^*^Tg/+^ mice ileums were isolated for qPCR on *iCre*, *TdT*, *Defa24, Defa21*, and *Mmp7*). **(H)** comparison of the number of Paneth cells observed in TdT*^Defa24iCre^*^Tg/+^ and TdT*^Defa6iCre^*^Tg/Tg^ mice. **(I)** Fold increase (FI) of iCre expression in different organs (ileum, colon, kidney, liver, lung, stomach) from TdT*^Defa24iCre^*^Tg/+^ versus TdT*^Defa24iCre^*^+/+^ mice (*n* = 3). **(C–I)***P*-values were analyzed with one-way ANOVA followed with *post hoc*, all *P*-values shown are from *post-hoc* tests (ns.: ANOVA or *post-hoc* test not significant, *P ≤ 0.05, ****P ≤ 0.0001). Each individual data point represents an individual mouse. All bars represent mean ± SEM.

The new transgenic line, having one *iCre* allele as well as one R26 floxed STOP TdT allele, *Defa24iCre*^Tg/+^ TdT^Tg/+^ (in short TdT*^Defa24iCre^*^Tg/+^) was compared with the *Defa6iCre*^Tg/+^ TdT^Tg/+^ (in short TdT*^Defa6iCre^*^Tg/+^) for intensity and Paneth cell specificity of TdT expression, and thus *iCre* expression. TdT^Tg/+^ mice without *iCre* were applied as controls. Ileum was isolated from 8-week-old mice, and tissue sections were stained with DAPI and for TdT and LYZ1. In **Figure 3A**, the crypt-specific signals of TdT in both lines are clearly evident in the representative sections. As expected, the colocalization of both LYZ and TdT signals provides confidence that the target cells are correctly identified. Zooming in on crypts shown in the full ileum image of **Figure 3A** fully confirms this: a clear co-localization of Lyz1 and TdTomato signal in both TdT*^Defa24iCre^*^Tg/+^ and TdT*^Defa6iCre^*^Tg/+^ mice is apparent (**Figure 3B**). However, these IHC data do not allow qualitative quantification. To perform quantitative comparisons, we isolated ileum samples of both transgenic and control lines, and qPCR was performed for *iCre* and *TdT* mRNA. *iCre* and *TdT* were indeed significantly more strongly expressed in the new transgenic line compared to the TdT*^Defa6iCre^*^Tg/+^ reference (**Figures 3C, D**). As expected, no changes were observed in other endogenous genes, such as *Defa24*, *Defa21*, and *Mmp7* (**Figures 3E–G**). To exclude that any observed effects were due to a difference in the number of PCs per crypt, we counted and compared the PC numbers per crypt in the Defa6 and Defa24 *Cre* transgenic mice based on TdT and LYZ1 signals. No differences could be detected in the number of cells per crypt positive for TdT and LYZ1 in the TdT*^Defa6iCre^*^Tg/Tg^*versus* TdT*^Defa24iCre^*^Tg/+^ (**Figure 3H**). To ensure Paneth cell specificity of the *iCre* expression in the TdT*^Defa24iCre^*^Tg/+^ line, we quantified the expression of the *iCre* gene in other organs (lung, liver, stomach, colon, and kidney, *n* = 3) and found the fold change between transgenic and wild-type mice was significantly higher in the ileum compared to all other tissues (**Figure 3I**).

### 3.7 Paneth cell–deficient mice by transgenic means

Our major ambition was to generate Paneth cell-deficient mice by cell ablation using a cross of Paneth cell–specific *Cre* mice with conditional DTA-expressing mice (Rosa26-promoter-floxed-STOP-DTA mice). Previous crosses using the *Defa6iCre* mice had been unsuccessful, even by breeding the *Cre* to homozygosity and having two *iCre* alleles (DTA*^Defa6iCre^*^Tg/Tg^ mice). Therefore, we generated DTA*^Defa24iCre^*^Tg/+^ mice and compared them with DTA*^Defa6iCre^*^Tg/Tg^ mice as well as with DTA^Tg/+^ mice, which are mice having no *Cre* alleles at all, and hence no DTA expression.

**Figure 4:**
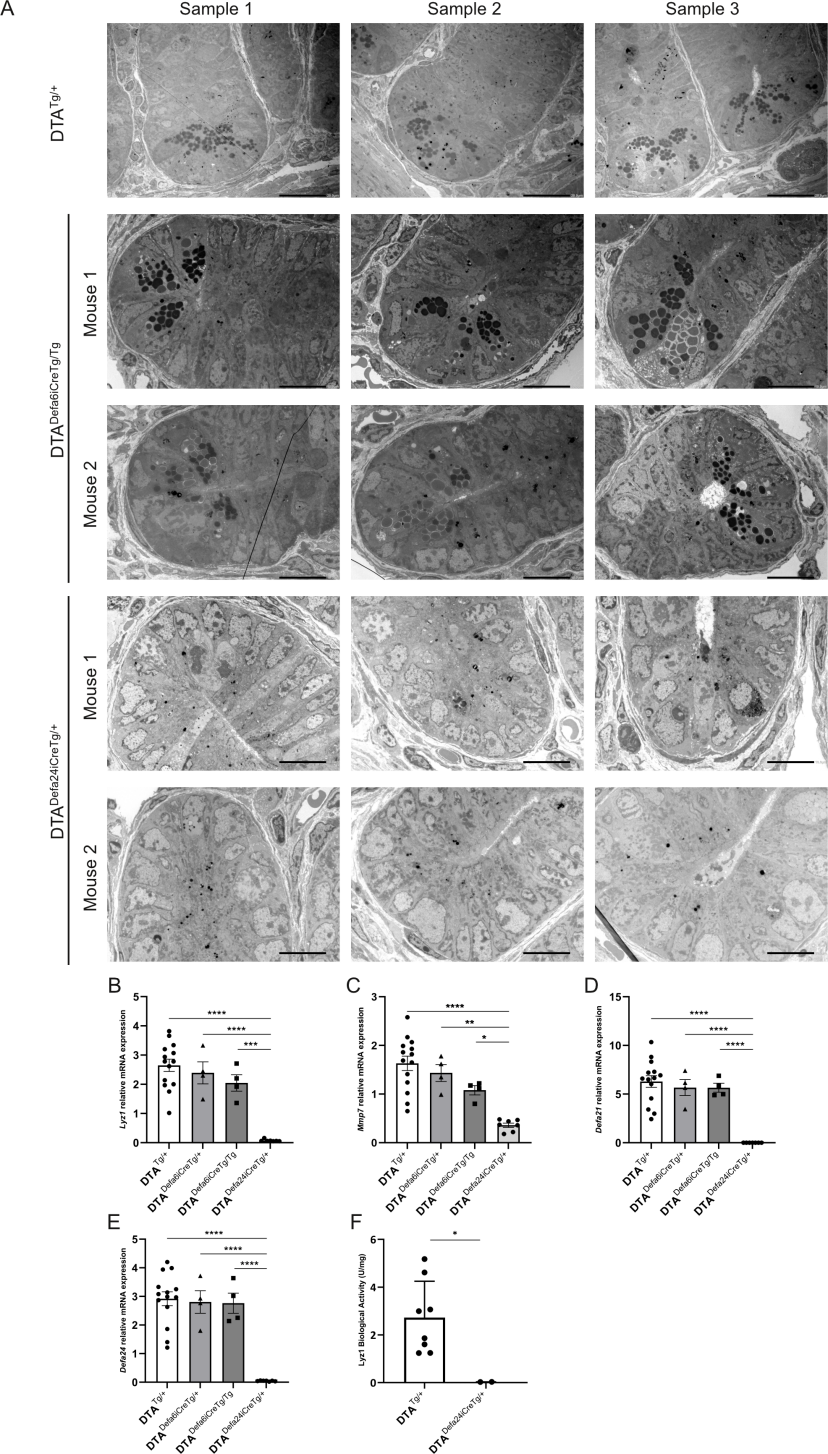
Paneth cell ablation using DTA*^Defa24iCre^*^Tg/+^ and DTA*^Defa6iCre^*^Tg/Tg^ mice. **(A)** TEM images of crypts of DTA^Tg/+^ mice (500×), DTA*^Defa24iCre^*^Tg/+^ (1000×), and DTA*^Defa6iCre^*^Tg/Tg^ mice (1000×), the scale bare represents 20 µm in 500× images and 10µm in 1000× images. **(B–E)** qPCR analysis of ileum samples of the DTA^Tg/+^ mice, DTA*^Defa24iCre^*^Tg/+^, DTA*^Defa6iCre^*^Tg/+,^and DTA*^Defa6iCre^*^Tg/Tg^ mice, measuring Paneth cell–specific transcripts, *Lyz1***(B)**, *Mmp7***(C)**, *Defa21***(D)**, and *Defa24***(E)**. **(F)** LYZ1 activity in ileum samples measured via LYZ1 activity assay kit in DTA^Tg/+^ and DTA*^Defa24iCre^*^Tg/+^ mice (*n* = 8 and 3, respectively). *P*-values were analyzed with one-way ANOVA **(B–F)** (*≤ ≤ 0.05, **P ≤ 0.01, ***P ≤ 0.001, ****P ≤ 0.0001). Each individual data point represents an individual mouse. All bars represent mean ± SEM.

Initially, we performed H&E and AB/PAS staining to visualize the Paneth cells. However, as these cells are often difficult to distinguish unambiguously by optical means, we used transmission electron microscopy instead. With this method we can easily zoom in on the crypts and identify Paneth cells by the presence of electrodense granules. **Figure 4A** shows that in DTA mice without active iCre (DTA^Tg/+^), there are clearly Paneth cells present in the crypts. When the floxed-STOP-DTA mice are crossed with the *Defa6iCre* mice, it is clear that this does not lead to full removal of the Paneth cells, since we can still see cells that have crypts with electrodense granules. When using the *Defa24iCre*, no more cells with electrodense granules can be detected in any crypt, indicating that this iCre does indeed lead to full depletion of the Paneth cells in the mice.

We then applied qPCR of Paneth cell–specific genes to detect Paneth cell signals in ileum samples. *Lyz1*, *Defa21, Defa24*, and *Mmp7* are Paneth cell–specific genes in the small intestine that are reliably measured by qPCR. As shown in **Figures 4B–E**, *Lyz1*, *Mmp7*, *Defa21*, and *Defa24* expression was reduced in DTA*^Defa6iCre^*^Tg/Tg^ mice but still detectable, while in DTA*^Defa24iCre^*^Tg/+^ the signals were absent for all but *Mmp7*, which still showed strong reduction in DTA*^Defa24iCre^*^Tg/+^ mice compared to the other transgenic mice, confirming the absence of Paneth cells and suggesting a chronic killing of these cells thanks to the strong efficiency of the CRE and DTA expression.

Based on the TEM and qPCR analyses, the *Defa24iCre*-based DTA Paneth cell–ablated mice will be an interesting tool to study (patho)physiological aspects of the deficiency of these cells in mice in the future. We investigated a straightforward aspect of the functional consequences of the apparent complete loss of Paneth cells in DTA^Defa24iCreTg/+^ mice, focusing on their normal role in controlling bacterial populations. A lysozyme activity assay was performed, and the DTA*^Defa24iCre^*^Tg/+^ mice showed a complete loss of lysozyme activity (**Figure 4F**).

## 4 Discussion

In this paper, we describe the generation of a novel Paneth cell–specific *Cre* transgenic mouse, which we believe will be of significant interest to researchers studying these specialized cells (1). Since the discovery and introduction of site-specific recombinases (SSRs), with *Cre* recombinase and *loxP* site being the most widely used, generating cell-specific *Cre* transgenic mice has become highly valuable for a variety of purposes (31). The most obvious purposes are those leading to a cell-specific depletion of a genomic fragment marked by two *loxP* sites (floxed fragment), with two general applications (32). In the first part of a coding gene (exons, for example), it can be floxed, and cell-specific *Cre* (whether continuously expressed or induced at the transcriptional or post-transcriptional level, e.g., by tamoxifen) can delete the floxed fragment, leading to a cell-specific depletion of the normal transcript and, therefore, a cell-specific knockout. In the second application, a floxed STOP sequence prevents transgene expression when placed between a strong, ubiquitous promoter and the transgene. This STOP sequence typically consists of multiple regulatory elements, including transcriptional stop signals, translational stop codons, splice donor sites, and so forth. The ROSA26 promoter, a stable and ubiquitously active element first identified on mouse chromosome 6, is commonly used in this system (33). By crossing a mouse carrying a knocked-in transgene under the ROSA26 promoter—interrupted by a floxed STOP—with a cell-specific *Cre*-expressing mouse, the resulting double-mutant animals will undergo Cre-mediated excision of the STOP sequence specifically in *Cre*-expressing cells, enabling transgene expression only in those cells. This approach is generally performed using a single mutant allele of each (one *Cre* allele and one transgenic allele), as this is typically sufficient to achieve adequate transgene expression.

In search of a good Paneth cell–specific *Cre* mouse, we first evaluated the already described *Cre*-transgenic mice with Paneth cell specificity (1, 34). Selecting the appropriate *Cre* driver is crucial, as each of these new alleles carries its own advantages and limitations. Two different research groups have generated *Cre* knock-in mice in the coding region of the *Lyz1* gene on chromosome 10 (23, 24). *Lyz1* is strongly expressed in Paneth cells, but the destruction of one of the two alleles might lead to a reduction in *Lyz1* mRNA in these Paneth cells. In addition, the mouse cell atlas, a single-cell expression database for mice, shows that Lyz1 may be present in alveolar monocytes and alveolar type II cells (9). If true, then using this *Cre* mouse for Paneth cell ablation purposes will also deplete these lung populations, which is not optimal and might lead to off-target effects. The *Defa6*-driven *Cre* mice were generated first, based on the then known −6500 bp to +34 bp fragment of the cryptdin 2 sequence as described, and have been the gold standard of PC-specific *Cre* lines. The other Paneth-specific *Cre* transgenic mouse was generated, again by two groups, by knocking in the *Cre* gene under control of the *Defa4* gene, but in the 3’UTR, and bringing the *Cre* expression under the influence of *Defa4* without disrupting the coding sequence. This strategy is good, but however, it is unclear in which gene *Cre* was actually knocked in, because the *Defa4* gene no longer exists. From the paper, we believe it is the *Defa28* gene. This gene, though expressed in Paneth cells, is expressed more than 10,000 times less compared to the *Defa24* gene.

Here we studied the expression levels of genes in purified Paneth cells by RNA-seq, as well as the stability of the transcripts in changing pathophysiological conditions and in different regions of the small intestine, and we concluded that *Defa24* or *Defa30* would be suitable candidates for driving *Cre* expression. Both genes were among the most highly expressed in Paneth cells, with minimal transcriptional changes observed across different localizations and physiological states (**Tables 1**, **2**). Furthermore, there is no evidence suggesting that these genes are transcribed outside of Paneth cells in mice (9). We generated mice expressing the improved *Cre* gene (*iCre*) under control of the *Defa24* sequences in a BAC construct and generated mice by injection in pronuclei of zygotes. There are three advantages of using BACs in transgenic research: (i) compared to plasmids, BACs form fewer concatemers at the integration site; (ii) genes in BAC clones are also less influenced by expression-repressive signals from the integration site because the BAC sequences function as insulators; and (iii) the *iCre* gene will have all *Defa24*-specific signals for correct expression that are present in a stretch of 41 kB, which is 20 kB before the *iCre* gene and 21 kB behind it. This provides an advantage over the previous Defa6-driven line, which only includes a limited upstream region of the gene. Our approach is much more likely to include a full set of regulator sequences. One of the four founders was selected and appeared to have stronger activity than the *Defa6iCre* transgenic mice, yet was Paneth cell specific.

Moreover, the transgenic line, when crossed with conditional DTA mice (as described above, using the R26 promoter and floxed STOP signals), led to ablation of all Paneth cells, which was also superior compared to the *Defa6iCre* line. We were especially interested in using this system to express a toxic gene, DTA, in Paneth cells and to generate Paneth cell–ablated mice, in which we have succeeded using heterozygous DTA*^Defa24iCre^*. Generation of a Paneth cell–deficient mouse will be an exceptional tool to study intestinal homeostasis, immunity, and host-microbe interactions (1, 2, 35). Of particular interest is the crosstalk between Paneth cells and intestinal stem cells in the context of intestinal turnover and remodeling. While Paneth cells play a unique role in providing niche factors essential for stem cell maintenance, the precise mechanisms by which the intestinal stem cell niche is preserved in their absence remain unclear (36). In 1997, Garabedian et al. already described mice generated for that purpose, but they were not designed to operate via *Cre*-mediated SSR (16). Rather, the mice consisted of DTA, cloned behind a so-called promoter of the mouse *cryptdin-2* gene, and the construct integrated randomly in the genome of mice. Though the mice displayed 82% Paneth cell ablation, the strategy is, in a way, a dead end, because the transgenic product is usable only for one purpose and does not have the versatility of a good *Cre* transgenic mouse. In this regard, the *Defa24iCre* mouse offers a distinct advantage for Paneth cell ablation, particularly when compared to previous methods involving dithizone, as it eliminates the potential off-target effects on both the host and the microbial community (19). Functional studies must be done with this new transgenic line in order to assess the impact of Paneth cell ablation on the overall intestinal homeostasis. Furthermore, it may also be interesting to profile the performance (efficiency) of our new *iCre* line for creating very large deletions such as *Apc*^+/^*^fle1–15^* (37).

Multiple updates to the α-defensin (*Defa*) locus have caused confusion among researchers, as its annotation has evolved over time (3), ultimately leading to the depletion of the *Defa6* gene after it was shown to be identical to the *Defa24* gene. The new transgenic line that we have generated, therefore, is another version of the *Defa6iCre* line generated by Adolph et al. (21). Yet it proves to be more active in expression and activity in Paneth cells (21). A reason for the apparent discrepancy of *Cre* expression in two models may be due to the technology that was applied. In contrast to Adolph et al. (21), we have applied a BAC-based system, which clearly has advantages over a plasmid system (as discussed above). Moreover, the integration site of the construct of Adolph and ours will surely be different, but ours may be favorable for expression of the *iCre* gene and protein.

This research underscores the value of the newly developed transgenic mouse for optimizing experimental strategies in Paneth cell-conditional gene manipulation. Considering the four key aspects of *Cre* gene targeting (specificity, leakiness, efficiency, and off-target effects), the *Defa24iCre* line demonstrates clear advantages over the previous version, the *Defa6iCre*.

## Data Availability

RNA-seq data deposited at the National Center for Biotechnology Information Gene Expression Omnibus public database (http://www.ncbi.nlm.nih.gov/geo/) under following accession numbers:Paneth cells sorted from wild-type C57BL/6J mice, (n=3): GSE269510 (**Supplementary Table 1**). Paneth cells from C57BL/6J cells, either injected with 250 \uf06dl PBS or injected with 25 \uf06dg recombinant TNF (n=4): GSE267790 (**Table 1**). Paneth cells sorted from wild-type C57BL/6J mice, (n=4), namely from the duodenum, the jejunum and the ileum: GSE255507 (**Table 2**).

## Supplementary material

Supplementary Figure 1 Graphical representation of the cloning strategy of the transgenic construct. For details, see Materials and Methods.

Supplementary Table 1 We are displaying a typical example of a bulk RNA-seq experiment from pure Paneth cells, sorted from wild-type C57BL/6J mice. Genes are ranked from the highest to the lowest detectable transcripts, that is, 13,130 different transcripts. *Lyz1* and some of the (correctly annotated) *Defa* genes are found on top of the list of most abundant mRNAs.

Supplementary Table 2 Primer sequences used for genotyping.

Supplementary Table 3 Primer sequences used for RT-qPCR.

## References

[1] Wallaeys C Garcia-Gonzalez N Libert C . Paneth cells as the cornerstones of intestinal and organismal health: a primer. EMBO Mol Med. (2022) 15:e16427. doi: 10.15252/emmm.202216427, PMID: 36573340 PMC9906427

[2] Lueschow SR McElroy SJ . The paneth cell: the curator and defender of the immature small intestine. Front Immunol. (2020) 11:587. doi: 10.3389/fimmu.2020.00587, PMID: 32308658 PMC7145889

[3] Timmermans S Wallaeys C De Beul S Garcia-Gonzales N Libert C . Detection of chimeric alpha-defensin transcripts and peptides in mouse Paneth cells. Front Immunol. (2025) 16:1543059. doi: 10.3389/fimmu.2025.1543059, PMID: 39981239 PMC11840258

[4] Timmermans S Wallaeys C Garcia-Gonzalez N Pollaris L Saeys Y Libert C . Identification and characterization of multiple paneth cell types in the mouse small intestine. Cells. (2024) 13:1435. doi: 10.3390/cells13171435, PMID: 39273007 PMC11394207

[5] Wallaeys C Garcia-Gonzalez N Timmermans S Vandewalle J Vanderhaeghen T De Beul S . Paneth cell TNF signaling induces gut bacterial translocation and sepsis. Cell Host Microbe. (2024) 32:1725–1743.e7. doi: 10.1016/j.chom.2024.08.007, PMID: 39243761 PMC12938039

[6] Sato T van Es JH Snippert HJ Stange DE Vries RG van den Born M . Paneth cells constitute the niche for Lgr5 stem cells in intestinal crypts. Nature. (2011) 469:415–8. doi: 10.1038/nature09637, PMID: 21113151 PMC3547360

[7] Verhagen MP Joosten R Schmitt M Välimäki N Sacchetti A Rajamäki K . Non-stem cell lineages as an alternative origin of intestinal tumorigenesis in the context of inflammation. Nat Genet. (2024) 56:1456–67. doi: 10.1038/s41588-024-01801-y, PMID: 38902475 PMC11250264

[8] Schmitt M Schewe M Sacchetti A Feijtel D van de Geer WS Teeuwssen M . Paneth cells respond to inflammation and contribute to tissue regeneration by acquiring stem-like features through SCF/c-kit signaling. Cell Rep. (2018) 24:2312–2328.e7. doi: 10.1016/j.celrep.2018.07.085, PMID: 30157426

[9] Han X Wang R Zhou Y Fei L Sun H Lai S . Mapping the mouse cell atlas by microwell-seq. Cell. (2018) 172:1091–1107.e17. doi: 10.1016/j.cell.2018.02.001, PMID: 29474909

[10] Fei L Chen H Ma L E W Wang R Fang X . Systematic identification of cell-fate regulatory programs using a single-cell atlas of mouse development. Nat Genet. (2022) 54:1051–61. doi: 10.1038/s41588-022-01118-8, PMID: 35817981

[11] Han X Zhou Z Fei L Sun H Wang R Chen Y . Construction of a human cell landscape at single-cell level. Nature. (2020) 581:303–9. doi: 10.1038/s41586-020-2157-4, PMID: 32214235

[12] Hall B Limaye A Kulkarni AB . Overview: generation of gene knockout mice. Curr Protoc Cell Biol. (2009). doi: 10.1002/0471143030.cb1912s44, PMID: 19731224 PMC2782548

[13] Kim H Kim M Im S-K Fang S . Mouse Cre-LoxP system: general principles to determine tissue-specific roles of target genes. Lab Anim Res. (2018) 34:147–59. doi: 10.5625/lar.2018.34.4.147, PMID: 30671100 PMC6333611

[14] Li S Chen L Peng X Wang C Qin B Tan D . Overview of the reporter genes and reporter mouse models. Anim Model Exp Med. (2018) 1:29–35. doi: 10.1002/ame2.12008, PMID: 30891544 PMC6357428

[15] Takahashi N Vanlaere I de Rycke R Cauwels A Joosten LAB Lubberts E . IL-17 produced by Paneth cells drives TNF-induced shock. J Exp Med. (2008) 205:1755–61. doi: 10.1084/jem.20080588, PMID: 18663129 PMC2525583

[16] Garabedian EM Roberts LJJ McNevin MS Gordon JI . Examining the role of paneth cells in the small intestine by lineage ablation in transgenic mice*. J Biol Chem. (1997) 272:23729–40. doi: 10.1074/jbc.272.38.23729, PMID: 9295317

[17] Sawada M Takahashi K Sawada S Midorikawa O . Selective killing of Paneth cells by intravenous administration of dithizone in rats. Int J Exp Pathol. (1991) 72:407–21. PMC2001955 1883741

[18] Sherman MP Bennett SH Hwang FFY Sherman J Bevins CL . Paneth cells and antibacterial host defense in neonatal small intestine. Infect Immun. (2005) 73:6143–6. doi: 10.1128/IAI.73.9.6143-6146.2005, PMID: 16113336 PMC1231051

[19] Berger JN Gong H Good M McElroy SJ . Dithizone-induced Paneth cell disruption significantly decreases intestinal perfusion in the murine small intestine. J Pediatr Surg. (2019) 54:2402–7. doi: 10.1016/j.jpedsurg.2019.02.021, PMID: 30857731 PMC6707906

[20] Vaishnava S Behrendt CL Ismail AS Eckmann L Hooper LV . Paneth cells directly sense gut commensals and maintain homeostasis at the intestinal host-microbial interface. Proc Natl Acad Sci. (2008) 105:20858–63. doi: 10.1073/pnas.0808723105, PMID: 19075245 PMC2603261

[21] Adolph TE Tomczak MF Niederreiter L Ko H-J Böck J Martinez-Naves E . Paneth cells as a site of origin for intestinal inflammation. Nature. (2013) 503:272–6. doi: 10.1038/nature12599, PMID: 24089213 PMC3862182

[22] Burger E Araujo A López-Yglesias A Rajala MW Geng L Levine B . Loss of Paneth cell autophagy causes acute susceptibility to Toxoplasma gondii-mediated inflammation. Cell Host Microbe. (2018) 23:177–190.e4. doi: 10.1016/j.chom.2018.01.001, PMID: 29358083 PMC6179445

[23] van Es JH Wiebrands K López-Iglesias C van de Wetering M Zeinstra L van den Born M . Enteroendocrine and tuft cells support Lgr5 stem cells on Paneth cell depletion. Proc Natl Acad Sci. (2019) 116:26599–605. doi: 10.1073/pnas.1801888117, PMID: 31843916 PMC6936398

[24] Yu S Tong K Zhao Y Balasubramanian I Yap GS Ferraris RP . Paneth cell multi-potency induced by Notch activation following injury. Cell Stem Cell. (2018) 23:46–59.e5. doi: 10.1016/j.stem.2018.05.002, PMID: 29887318 PMC6035085

[25] Wang R Zhang P Wang J Ma L E W Suo S . Construction of a cross-species cell landscape at single-cell level. Nucleic Acids Res. (2023) 51:501–16. doi: 10.1093/nar/gkac633, PMID: 35929025 PMC9881150

[26] Balasubramanian I Bandyopadhyay S Flores J Bianchi-Smak J Lin X Liu H . Infection and inflammation stimulate expansion of a CD74+ Paneth cell subset to regulate disease progression. EMBO J. (2023) 42:e113975. doi: 10.15252/embj.2023113975, PMID: 37718683 PMC10620768

[27] Madisen L Zwingman TA Sunkin SM Oh SW Zariwala HA Gu H . A robust and high-throughput Cre reporting and characterization system for the whole mouse brain. Nat Neurosci. (2010) 13:133–40. doi: 10.1038/nn.2467, PMID: 20023653 PMC2840225

[28] Lee P Morley G Huang Q Fischer A Seiler S Horner JW . Conditional lineage ablation to model human diseases. Proc Natl Acad Sci U.S.A. (1998) 95:11371–6. doi: 10.1073/pnas.95.19.11371, PMID: 9736743 PMC21649

[29] Cardenas-Diaz FL Liberti DC Leach JP Babu A Barasch J Shen T . Temporal and spatial staging of lung alveolar regeneration is determined by the grainyhead transcription factor Tfcp2l1. Cell Rep. (2023) 42:112451. doi: 10.1016/j.celrep.2023.112451, PMID: 37119134 PMC10360042

[30] Nagy A . Cre recombinase: the universal reagent for genome tailoring. Genesis. (2000) 26:99–109. doi: 10.1002/(SICI)1526-968X(200002)26:2<99::AID-GENE1>3.0.CO;2-B, PMID: 10686599

[31] Kühn R Schwenk F Aguet M Rajewsky K . Inducible gene targeting in mice. Science. (1995) 269:1427–9. doi: 10.1126/science.7660125, PMID: 7660125

[32] Rajewsky K Gu H Kühn R Betz UA Müller W Roes J . Conditional gene targeting. J Clin Invest. (1996) 98:600–3. doi: 10.1172/JCI118828, PMID: 8698848 PMC507466

[33] Zambrowicz BP Imamoto A Fiering S Herzenberg LA Kerr WG Soriano P . Disruption of overlapping transcripts in the ROSA βgeo 26 gene trap strain leads to widespread expression of β-galactosidase in mouse embryos and hematopoietic cells. Proc Natl Acad Sci U.S.A. (1997) 94:3789–94. doi: 10.1073/pnas.94.8.3789, PMID: 9108056 PMC20519

[34] Parry L Young M El Marjou F Clarke AR . Protocols for analyzing the role of paneth cells in regenerating the murine intestine using conditional cre-lox mouse models. J Vis Exp. (2015) 105:53429. doi: 10.3791/53429, PMID: 26649885 PMC4755722

[35] Barreto E Barreto L Rattes IC da Costa AV Gama P . Paneth cells and their multiple functions. Cell Biol Int. (2022) 46:701–10. doi: 10.1002/cbin.11764, PMID: 35032139

[36] Quintero M Samuelson LC . Paneth cells: dispensable yet irreplaceable for the intestinal stem cell niche. Cell Mol Gastroenterol Hepatol. (2025) 19:101443. doi: 10.1016/j.jcmgh.2024.101443, PMID: 39708920 PMC11847746

[37] Cheung AF Carter AM Kostova KK Woodruff JF Crowley D Bronson RT . Complete deletion of Apc results in severe polyposis in mice. Oncogene. (2010) 29:1857–64. doi: 10.1038/onc.2009.457, PMID: 20010873 PMC2990498

