# Correction: Generation of a new Paneth cell-specific *Cre*-recombinase transgenic mouse line

**DOI:** 10.3389/fimmu.2025.1733126

**Published:** 2025-12-02

**Authors:** Natalia Garcia-Gonzalez, Charlotte Wallaeys, Wendy Toussaint, Tino Hochepied, Sylviane Dewaele, Somara De Beul, Robin Van Loke, Jolien Vandewalle, Steven Timmermans, Richard S. Blumberg, Claude Libert

**Affiliations:** 1 VIB Center for Inflammation Research, Ghent, Belgium; 2 Department of Biomedical Molecular Biology, Ghent University, Ghent, Belgium; 3 Division of Gastroenterology, Department of Medicine, Brigham and Women’s Hospital, Harvard Medical School, Boston, MA, United States

**Keywords:** Paneth cells, *Defa24iCre*, defensins, *Cre*-recombinase transgenic mouse model, *Cre/loxP*

Author “Richard S. Blumberg” was omitted as an author in the published article. The correct author list reads:

“Natalia Garcia-Gonzalez^1,2^, Charlotte Wallaeys^1,2^, Wendy Toussaint^1,2^, Tino Hochepied^1,2^, Sylviane Dewaele^1,2^, Somara De Beul^1,2^, Robin Van Loke^1,2^, Jolien Vandewalle^1,2^, Steven Timmermans^1,2^, Richard S. Blumberg^3^ and Claude Libert^1,2,*^”

“^1^VIB Center for Inflammation Research, Ghent, Belgium.

^2^Department of Biomedical Molecular Biology, Ghent University, Ghent, Belgium.

^3^Division of Gastroenterology, Department of Medicine, Brigham and Women’s Hospital, Harvard Medical School, Boston, MA, United States”

The **Author Contributions** statement has been corrected to read:

The original version of this article has been updated.

“NG-G: Conceptualization, Investigation, Methodology, Writing – original draft, Writing – review & editing, Data curation, Formal analysis, Visualization. CW: Conceptualization, Investigation, Methodology, Writing – original draft, Visualization, Formal analysis. WT: Conceptualization, Investigation, Methodology, Writing – original draft. TH: Conceptualization, Investigation, Methodology, Writing – original draft. SD: Investigation, Methodology, Writing – original draft. SDB: Investigation, Methodology, Writing – original draft. RV: Data curation, Methodology, Validation, Writing – review & editing. JV: Data curation, Methodology, Supervision, Formal analysis, Project administration, Funding acquisition, Writing – review & editing. ST: Data curation, Investigation, Software, Writing – original draft, Writing – review & editing, Formal analysis. RSB: Conceptualization, Funding acquisition, Investigation, Methodology, Project administration, Supervision, Validation CL: Conceptualization, Funding acquisition, Investigation, Methodology, Project administration, Supervision, Validation, Writing – original draft, Writing – review & editing, Formal analysis, Visualization.”

The funder “National Institute of Diabetes and Digestive and Kidney Diseases (NIDDK) at the National Institute of Health (NIH), R01DK088199 to Richard S. Blumberg” was erroneously omitted.

The original version of this article has been updated.

