# Supplementary figures and images for: Generation of a new Paneth cell–specific Cre-recombinase transgenic mouse line
Source: Front Immunol. 2025 Jun 25;16:1576995. doi: 10.3389/fimmu.2025.1576995 (PMC12237991; doi:10.3389/fimmu.2025.1576995)

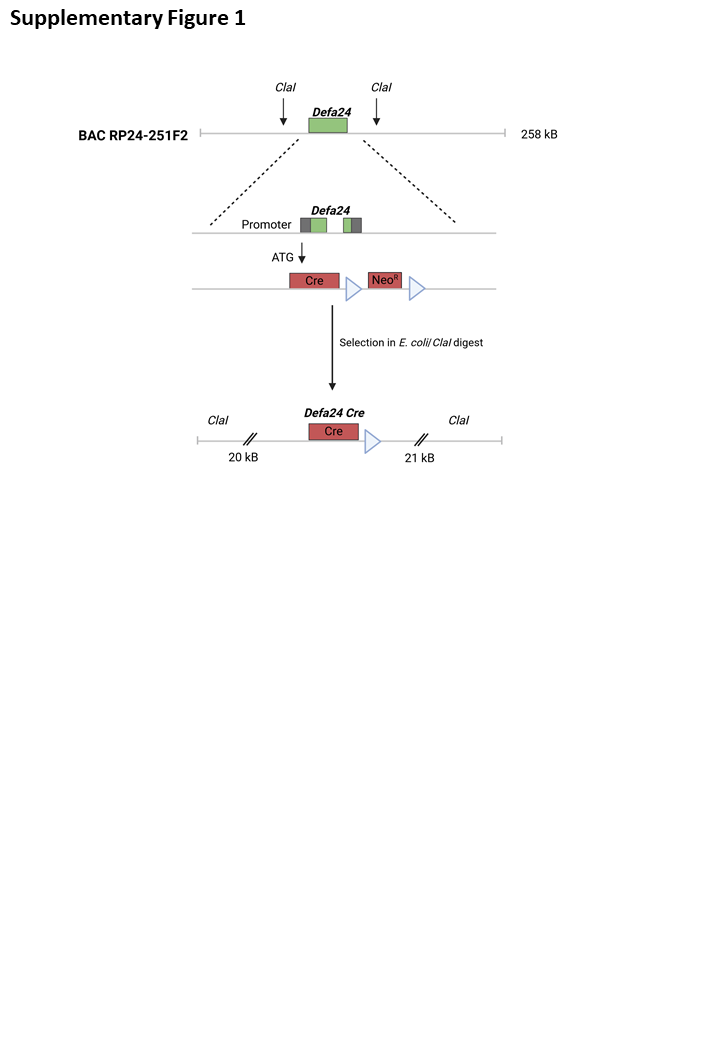

Supplement: Supplementary Figure 1 — Graphical representation of the cloning strategy of the transgenic construct. For details, see Materials and Methods. [file Image1.tif]
